# Single Nucleotide Polymorphisms in Cellular Drug Transporters Are Associated with Intolerance to Antiretroviral Therapy in Brazilian HIV-1 Positive Individuals

**DOI:** 10.1371/journal.pone.0163170

**Published:** 2016-09-20

**Authors:** Mônica Barcellos Arruda, Francine Campagnari, Tailah Bernardo de Almeida, José Carlos Couto-Fernandez, Amilcar Tanuri, Cynthia Chester Cardoso

**Affiliations:** 1 Laboratório de Virologia Molecular, Departamento de Genética, Instituto de Biologia, Universidade Federal do Rio de Janeiro, Rio de Janeiro, Brasil; 2 Deoxi Biotecnologia. Araçatuba, São Paulo, Brasil; 3 Laboratório de Aids e Imunologia Molecular, Instituto Oswaldo Cruz, Fiocruz, Rio de Janeiro, Brasil; University of Rochester Medical Center, UNITED STATES

## Abstract

Adverse reactions are the main cause of treatment discontinuation among HIV+ individuals. Genes related to drug absorption, distribution, metabolism and excretion (ADME) influence drug bioavailability and treatment response. We have investigated the association between single nucleotide polymorphisms (SNPs) in 29 ADME genes and intolerance to therapy in a case-control study including 764 individuals. Results showed that 15 SNPs were associated with intolerance to nucleoside and 11 to non-nucleoside reverse transcriptase inhibitors (NRTIs and NNRTIs), and 8 to protease inhibitors (PIs) containing regimens under alpha = 0.05. After Bonferroni adjustment, two associations remained statistically significant. SNP rs2712816, at *SLCO2B1* was associated to intolerance to NRTIs (OR_GA/AA_ = 2.37; p = 0.0001), while rs4148396, at *ABCC2*, conferred risk of intolerance to PIs containing regimens (OR_CT/TT_ = 2.64; p = 0.00009). Accordingly, haplotypes carrying rs2712816A and rs4148396T alleles were also associated to risk of intolerance to NRTIs and PIs, respectively. Our data reinforce the role of drug transporters in response to HIV therapy and may contribute to a future development of personalized therapies.

## Introduction

The infection by HIV-1 is a pandemic condition that affects nearly 37 million people worldwide [1]. The treatment of HIV positive individuals is based on the combined antiretroviral therapy (cART), which consists on the administration of three antiretrovirals (ARVs) from at least two different classes. These classes include the nucleoside reverse transcriptase inhibitors (NRTIs), non-nucleoside reverse transcriptase inhibitors (NNRTIs), protease inhibitors (PIs), fusion and entry inhibitors, and integrase inhibitors. Introduction of HAART in 1996 has led to clear health improvements to the HIV+ individuals, with reduced morbidity and increase in life expectancy [2]. However, the emergence of drug resistance and the development of adverse reactions to the ARVs are still of major concern since they may impair therapy effectiveness.

Adverse reactions (ARs) are one of the most important factors associated with reduced life quality among individuals undergoing antiretroviral therapy. In fact, ARs have been reported as the most common cause of treatment discontinuation and changes in cART regimen [3–6]. Gastrointestinal intolerance, anemia and hypersensitivity reactions are commonly observed since the early beginning of the treatment. The long-term use of the ARVs may also lead to metabolic disorders, including lipodystrophy and dyslipidemia, and peripheral neuropathies [5–7]. Moreover, ARs can also limit treatment effectiveness by reducing adherence [8–11] and, consequently, favoring the emergence of drug resistant viruses.

Variations in genes coding for molecules involved in drug absorption, distribution, metabolism and excretion (ADME) have been consistently associated to both short-term and long-term adverse reactions to the ARVs. The impact of such variations is clearly reflected by the interindividual variations in antiretroviral plasma concentrations after taking the same dosage. Therefore, tolerance to treatment can also be substantially variable. Although much remains unknown, there is extensive data regarding the influence of *ABCB1* variations and plasma or intracellular levels of protease inhibitors [12]. Similar results have been observed for a second ABC transporter, coded by *ABCC2*. In addition, single nucleotide polymorphisms (SNPs) and haplotypes of *CYP2B6* have been consistently associated to efavirenz clearance and also to central nervous system adverse reactions to this drug [13–15]. Moreover, atazanavir-associated hyperbilirubinemia was associated to a microsatellite at *UGT1A1* promoter [16]. Mitochondrial DNA haplogroups and variations in genes coding for inflammatory mediators and apolipoproteins have also been suggested as predictors for lipid disorders [17–19].

According to recent estimates, there are about 781,000 people living with HIV in Brazil, and 405,000 are currently undergoing treatment [20]. However, just a few studies have been conducted to describe the role of host genetics in response to cART in our population [16, 21–25]. Therefore, the prevalence of SNPs in ADME genes among Brazilian HIV positive individuals remains largely unknown.

The present study was designed to investigate the association between 346 SNPs in 29 ADME genes and intolerance to antiretroviral therapy among Brazilians. For this purpose, 764 individuals undergoing antiretroviral therapy were enrolled in a case-control study. Our data showed a clear association between *ABCC2* SNPs and treatment modification due to intolerance to protease inhibitors, while *SLCO2B1* genetic variations increased the risk of intolerance to NRTIs.

## Methods

### Ethics Statement

The present study was approved by Fiocruz Institutional Review Board (IRB) and a written informed consent was obtained from all subjects. All analyses were conducted according to the principles expressed in the Declaration of Helsinki.

#### Subjects and study design

A total of 764 HIV-1+ individuals were enrolled in this study. All samples were obtained from the biorepository of the AIDS and Molecular Immunology Laboratory at Fiocruz, Rio de Janeiro, Brazil. This laboratory is a part of the Brazilian Network for HIV-1 Genotyping (RENAGENO), which was implemented in 2001 with the aim of providing free HIV genotyping tests for patients in HAART failure [26]. Patients from both genders, born in Rio de Janeiro and with a minimum age of 18 years old were considered eligible. A patient was included in the study if he/she had: (1) a complete history of cART schemes since first line therapy available in the file, (2) *buffy coat* samples available for DNA extraction. Patients under treatment for hepatitis or tuberculosis and women who exhibited adverse reactions during pregnancy were excluded from the study. The patients who developed adverse reactions to any antiretroviral were classified as cases (N = 359), while the control group (N = 405) included patients to whom the treatment was safe. Considering a minor allele frequency of 0.05 and alpha = 0.0001 (Bonferroni adjustment for 346 candidate SNPs), the minimum OR value to reach 80% of power with this sample size was 2 under an additive model.

A subject was not included in the control group if he/she has not been treated for at least 6 months. A total of 195 controls (48%) were still undergoing first-line therapy (first scheme) at the moment of data collection, with a medium time of 3.9 ± 2 years using the same drug combination. For those who had already changed treatment, the medium time of permanence in each scheme was 2.87 ± 1.87 years.

Clinical description of specific adverse reactions was available for a limited number of subjects (N = 65 for cases of intolerance to nucleoside reverse transcriptase inhibitors, 26 for non-nucleoside reverse transcriptase inhibitors, and 32 for protease inhibitors). Therefore, the outcome “intolerance” was used for case definition. Among the cases of intolerance to NRTIs, 31 developed anemia attributed to zidovudine and 10 exhibited lipodistrophy due to stavudine, which was also associated to peripheral neuropathy in 9 individuals. Fifteen individuals developed dyslipidemia and the remaining 8 cases exhibited rash, gastrointestinal adverse effects and lactic acidosis (N = 3, 3 and 2, respectively). Among the subjects who developed intolerance to NNRTIs, the most prevalent adverse reaction was cutaneous rash after nevirapine use (N = 16), followed by neuropsychiatric disorders in response to efavirenz (N = 7) and gastrointestinal disorders (N = 3). In the group of intolerance to protease inhibitors, gastrointestinal adverse reactions including diarrhea and nausea due to lopinavir/ritonavir use were the most prevalent (N = 11), followed by nephrolithiasis due to indinavir (N = 9). Lipodistrophy and dyslipidaemia were observed in 5 and 3 cases, respectively. The remaining 4 cases exhibited jaundice due to atazanavir and cutaneous rash (N = 2, each).

First of all, an overall analysis was developed including all cases and controls (“All ARVs”), regardless the drug class. Afterwards, three different case-control studies were conducted to characterize association between pharmacogenetic markers and intolerance to: (1) nucleoside reverse transcriptase inhibitors (NRTIs), (2) non- nucleoside reverse transcriptase inhibitors (NNRTIs) or (3) protease inhibitors (PIs). For this analysis, all individuals exhibiting adverse reactions to drugs belonging to the same class were grouped in a single case-control.

Genetic ancestry was determined using a panel of 28 ancestry informative SNPs previously validated for Brazilian population [27]. Distribution of each gender and proportions of European and African ancestries are represented in Table 1.

**Table 1:** General characteristics of the subjects of the entire sample and according to the antiretroviral classes.

	ALL ARVs		NRTIs		NNRTIs		PIs	
	Cases	Controls	Cases	Controls	Cases	Controls	Cases	Controls
**N**	359	405	141	402	99	297	115	203
**Gender**								
Male	222 (0.62)	259 (0.64)	97 (0.69)	257 (0.64)	62 (0.63)	186 (0.63)	70 (0.61)	138 (0.68)
Female	137 (0.38)	146 (0.36)	44 (0.31)	145 (0.36)	37 (0.37)	111 (0.37)	45 (0.39)	65 (0.32)
**Genetic ancestry** ^a^								
African	0.345 ± 0.242	0.397 ± 0.249	0.344 ± 0.231	0.397 ± 0.248	0.356 ± 0.242	0.395 ± 0.253	0.326 ± 0.245	0.381 ± 0.233
European	0.655 ± 0.241	0.602 ± 0.249	0.656 ± 0.231	0.603 ± 0.248	0.644 ± 0.253	0.605 ± 0.241	0.674 ± 0.233	0.619 ± 0.245

Data are represented as N (frequency) for gender and mean ± standard deviation for proportions of African and European genetic ancestries. ARVs = antiretrovirals. NRTIs = nucleoside reverse transcriptase inhibitors; NNRTIs = non-nucleoside reverse transcriptase inhibitors; PIs = protease inhibitors.

^a^ p < 0.05 for comparisons between cases and controls for ALL ARVs and NRTIs group.

### Single Nucleotide Polymorphisms (SNPs) Selection

Selection of candidate genes and SNPs was based mainly on literature search, as a way to replicate previous associations. We have also searched for additional SNPs in coding and regulatory regions of all candidate genes using the SNPper tool. In order to increase gene coverage, *tag* SNPs were selected from HapMap data bank using the following parameters: minor allele frequency of 0.05 in CEU (Utah residents with Northern and Western European ancestry) or YRI (Yoruba in Ibadan, Nigeria) populations and r^2^ cutoff of 0.8. Following this strategy, 346 SNPs in 29 candidate genes were selected (S1 Table).

### DNA Extraction and SNP Genotyping

Genomic DNA was obtained from *buffy coat* samples using the QIAamp^®^ DNA Blood Mini Kit (QIAGEN) according to manufacturer’s instructions. A total of 345 SNPs was genotyped using Illumina Golden Gate^®^ assays (Illumina, San Diego, CA, USA) according to the manufacturer’s instructions. Briefly, the assay includes two allele-specific oligos (ASO) and a locus-specific oligo (LSO) that hybridizes several bases downstream from the SNP site. The three oligonucleotide sequences contain regions of complementarity with universal PCR primer sites. DNA samples (250 ng) were activated and hybridized to the assay oligonucleotides designed for each SNP locus. Following hybridization, several wash steps were performed before base extension and ligation of the ASO and LSO primers. Amplification of the full length products was performed using Cy3- and Cy5-labeled universal PCR primers and Titanium^®^ DNA polymerase (CloneTech, Mountain View, CA, USA). Finally, Golden Gate^®^ assay products were hybridized to the BeadChip and the fluorescence was analyzed using an iScan instrument (Illumina, San Diego, CA, USA). Genotype calling was performed using the software Genome Studio (Illumina, San Diego, CA, USA). The SNP rs3745274, located at *CYP2B6* gene, did not pass Illumina criteria for assay design and, therefore, was genotyped using SNaPshot^®^ analysis (Thermo Scientific, Massachusetts, USA). Briefly, PCR amplification was performed using QIAGEN Multiplex PCR kit (QIAGEN, Hilden, Germany) and specific forward (5’-GATTGAACACCTACTCTGCCCAGCC-3’) and reverse (5’-AGACGATGGAGCAGATGATGTTGGC-3) primers at 0.2 μM. PCR products were cleaned using 3U of FastAP (Thermo Scientific, Massachusetts, USA) and 1U of Exonucelase I (Affimetrix, California, USA). SNaPshot reactions were performed using the SNaPshot™ Multiplex kit (Thermo Scientific, Massachusetts, USA) according to manufacturer’s instructions and the specific primer for rs3745274 (5’-CCCTCATGGACCCCACCTTCCTCTTCCA-3’) at 2 μM. SNaPshot products were treated with FastAP 1U and submitted to capillary electrophoresis on ABI3130 Genetic Analyzer (Thermo Scientific, Massachusetts, USA) using the standard fragment analysis protocol. Genotyping was performed using GeneMapper software, version 4.0 (Thermo Scientific, Massachusetts, USA)”.

### Statistical Analyses

Deviations from Hardy-Weiberg Equilibrium (HWE) were assessed by χ^2^ tests. Comparisons between cases and controls were performed using unconditional logistic regression models controlling for potential confounders such as gender, age at diagnosis and proportions of European and African ancestries. A stepwise logistic regression analysis was performed to select covariates. All SNPs were analyzed under dominant and codominant models. A Bonferroni adjustment was applied to avoid type I error. A Cochran–Armitage trend test was conducted to describe possible allele dose effects. Pairwise linkage disequilibrium (LD) patterns were determined using the r^2^ statistics and a cutoff of r^2^ ≥ 0.8 to define “tags”. SNPs with nominal p-values < 0.05 in both models (dominant and codominant) were included in haplotype analyses. Haplotype frequencies were estimated by maximum likelihood and compared using the same logistic regression models applied for isolated SNPs. All analyses were performed using the R for windows, version 2.14.1, with the packages ‘‘genetics”, “gap”, “SNPassoc”, ‘‘haplo.stats” and ‘‘coin”.

## Results

### SNPs in ADME Genes Are Associated with Intolerance to Different cART Regimens

A total of 346 SNPs were genotyped in our study. After quality control, 76 SNPs were excluded from analysis due to HWE deviations (N = 38) or because they were monomorfic (N = 38) in our control sample. The minor allele frequencies are shown for each SNP in S1 Table. The remaining 270 candidate SNPs were analyzed separately using logistic regression models adjusted for sex and genetic ancestry. First of all, patients were compared according to the development of adverse reactions to any antiretroviral (All ARVs), and a total of 12 SNPs were significantly associated to this outcome in both multivariate models (dominant and codominant adjusted for gender and genetic ancestry) under a 0.05 significance level (S2 Table). When statistical analyses were stratified according to the ARVs classes, 15 SNPs were associated to adverse reactions to NRTIs, 11 to NNRTIs and 8 to PIs (p < 0.05 in dominant and codominant models; S2 Table). After Bonferroni adjustment for multiple comparisons (270 SNPs; alpha = 0.0002), two associations remained statistically significant (S2 Table, Tables 2 and 3). The SNP rs2712816 (G>A) at *SLCO2B1* gene was associated to increased risk of adverse reactions to NRTIs (OR = 2.37; p = 0.0001 for GA/AA genotypes after adjustment for gender and genetic ancestry; S2 Table), while rs4148396 (C>T), located at *ABCC2*, conferred risk of intolerance to PIs (OR = 2.64; p = 0.00009 for CT/TT genotypes; S2 Table). OR values remained virtually the same when patients who developed adverse reactions to both NRTIs and PIs (N = 25) were removed from analysis (data not shown).

**Table 2:** Association between *SLCO2B1* gene and intolerance to nucleoside reverse transcriptase inhibitors.

Genotype/Haplotype	Controls ^a^	Cases ^a^	OR (_95%_CI)	OR (_95%_CI) ^b^
**SNP rs2712816**				
GG	171 (0.43)	32 (0.23)	Reference	Reference
GA	178(0.44)	80 (0.57)	2.40 (1.52–3.81)	2.29 (1.43–3.65)
AA	52 (0.13)	29 (0.20)	2.98 (1.65–5.38)	2.69 (1.46–4.95)
	401	141	p = 0.00006 ^c^	p = 0.0004 ^c^
**SNP rs949069**				
GG	146 (0.36)	73 (0.52)	Reference	Reference
GA	188 (0.47)	57 (0.40)	0.61 (0.40–0.91)	0.65 (0.43–0.98)
AA	67 (0.17)	11 (0.08)	0.33 (0.16–0.66)	0.34 (0.17–0.69)
	401	141	p = 0.0013 ^c^	p = 0.0032 ^c^
**SNP rs12422149**				
GG	296 (0.74)	119 (0.84)	Reference	Reference
GA	93 (0.23)	21 (0.14)	0.56 (0.33–0.94)	0.55 (0.33–0.93)
AA	13 (0.03)	1 (0.01)	0.19 (0.02–1.48)	0.18 (0.02–1.40)
	402	141	p = 0.0143 ^c^	p = 0.0104 ^c^
**SNP rs1676885**				
AA	298 (0.74)	88 (0.63)	Reference	Reference
AG	93 (0.23)	41 (0.29)	1.49 (0.96–2.31)	1.35 (0.86–2.12)
GG	11 (0.03)	11 (0.08)	3.39 (1.42–8.07)	3.19 (1.33–7.65)
	402	140	p = 0.0096 ^c^	p = 0.0251 ^c^
**Haplotypes rs2712816/rs949069/ rs12422149/rs1676885**				
**G/G/G/A**	0.24	0.21	Reference	Reference
**A/G/G/A**	0.23	0.30	1.61 (1.05–2.47; p = 0.03)	1.39 (1.01–1.93; p = 0.04)
**A/G/G/G**	0.11	0.18	1.81 (1.13–2.88; p = 0.01)	1.49 (1.02–2.15; p = 0.04)
**G/A/A/A**	0.13	0.07	0.65 (0.37–1.15; p = 0.14)	0.95 (0.64–1.38; p = 0.77)
**G/A/G/A**	0.24	0.19	0.96 (0.61–1.51; p = 0.84)	1.19 (0.85–1.64; p = 0.31)
**G/G/G/G**	0.01	0.03	3.46 (0.85–14.04; p = 0.08)	2.45 (0.61–9.83; p = 0.21)

OR = odds ratio; CI = confidence interval.

^a^ Results are shown as N (frequency) for SNP genotypes and frequencies estimated by maximum likelihood for haplotypes;

^b^ results adjusted for gender and genetic ancestry;

^c^ overall p-value for codominant model (2 degrees of freedom).

**Table 3:** Association between *ABCC2* gene and intolerance to protease inhibitors.

Genotype/Haplotypes	Controls ^a^	Cases ^a^	OR (95% CI; p)	OR (95% CI; p) ^b^
**SNP rs4148396**				
**CC**	110(54.2)	36(31.3)	Reference	Reference
**CT**	78(38.4)	62(53.9)	2.43 (1.47–4.02)	2.47 (1.47–4.13)
**TT**	15(7.4)	17(14.8)	3.46 (1.57–7.63)	3.68 (1.62–8.35)
	203	115	p = 0.0003 ^c^	p = 0.0003 ^c^
**SNP rs3740066**				
GG	115 (0.57)	48 (0.42)	Reference	Reference
GA	74 (0.36)	54 (0.47)	1.75 (1.08–2.84)	1.74 (1.06–2.87)
AA	14 (0.07)	13 (0.11)	2.22 (0.97–5.08)	2.27 (0.96–5.33)
	203	115	p = 0.032 ^c^	p = 0.04 ^c^
**Haplotypes rs4148396/ rs3740066**				
**C/G**	0.67274	0.54838	Reference	Reference
**C/A**	0.06125	0.03422	0.71 (0.30–1.69; p = 0.44)	0.78 (0.32–1.88; p = 0.58)
**T/A**	0.18998	0.31360	2.06 (1.38–3.06; p = 0.0004)	2.09 (1.37–3.17; p = 0.0006)
**T/G**	0.07603	0.10379	1.75 (0.94–3.28; p = 0.08)	1.92 (1.02–2.83; p = 0.0442)

OR = odds ratio; CI = confidence interval.

^a^ Results are shown as N (frequency) for SNP genotypes and frequencies estimated by maximum likelihood for haplotypes;

^b^ results adjusted for gender and genetic ancestry;

^c^ overall p-value for codominant model (2 degrees of freedom).

### Association between *SLCO2B1* and Intolerance to Nucleoside Reverse Transcriptase Inhibitors

In addition to rs2712816, three SNPs at *SLCO2B1* (rs12422149, rs1676885 and rs949069) were associated to adverse reactions to NRTIs at a 0.05 significance level (S2 Table). SNP rs1676885 was associated to increased risk of intolerance (adjusted OR = 1.55 for AG/GG), while results obtained for rs949069 (adjusted OR = 0.57 for GA/AA) and rs12422149 (adjusted OR = 0.5 for GA/AA) suggested a protective effect. In all cases, the results obtained suggested an allele-dose effect, with more prominent effects observed among homozygotes for the minor allele (Table 2). This trend was confirmed by a Cochran-Armitage test, which resulted in adjusted p-values of 0.0005 for rs2712816, 0.003 for rs949069 and 0.01 for rs12422149 and rs1676885.

Results of linkage disequilibrium (LD) analyses have showed low association between the four markers tested (r^2^ < 0.8). Therefore, all SNPs were included in haplotype analysis. Comparisons of haplotype frequencies in cases and controls corroborated the data obtained when SNPs were analyzed separately (Table 2). Indeed, both haplotypes carrying rs2712816**A** allele increased the susceptibility to adverse reactions to NRTIs when compared to the baseline rs2712816**G**/rs949069**G** /rs12422149**G** /rs1676885**A** (adjusted OR = 1.39 for **A**/G/G/A and 1.49 for **A**/G/G/G; p = 0.04 for both haplotypes).

### Association between *ABCC2* and Intolerance to Protease Inhibitors

Besides rs4148396, four other SNPs at *ABCC2* (rs2073337, rs2804398, rs2804400, rs3740066) were associated with intolerance to PIs (p < 0.05; S2 Table, Table 3). From these, rs2804398 and rs2804400 were in almost perfect linkage disequilibrium with rs4148396 (r^2^ > 0.9) while rs2073337 was in moderate/strong LD (r^2^ = 0.66). Therefore, rs4148396 was defined as a tag for the entire bin. SNP rs3740066 was also included in the following analyses, since the low LD patterns with rs4148396 (r^2^ = 0.37) indicate that this variation belongs to an independent bin. The results obtained from both SNPs indicated an association with increased risk of intolerance to PIs containing regimens (adjusted OR = 2.64 for rs4148396 T carriers and 1.82 for rs3740066 A carriers; S2 Table). Moreover, the OR values suggested that the homozygotes were at higher risk of developing intolerance to PIs as compared to the heterozygotes (3.68 *vs* 2.47 for rs4148396 and 2.27 *vs* 1.74 for rs3740066; Table 3), suggesting an allele dose effect. This trend was confirmed by a Cochran-Armitage trend test (p = 0.0002 for rs4148396 and 0.03 for rs3740066).

Next, rs4148396 and rs3740066 alleles were combined in haplotypes. After analysis, the combination of rs4148396**T**/rs3740066**A** was associated to increased risk of adverse reactions to PIs (adjusted OR = 2.09; p = 0.0006), as compared to the baseline rs4148396**C**/rs3740066**G**, reinforcing the risk effect observed for rs4148396**T** and rs3740066**A** in single SNP analysis (Table 3).

## Discussion

In this study, we have investigated the association between SNPs in ADME genes and intolerance to cART regimens. Our results showed a consistent association between *SLCO2B1* gene variations and intolerance to NRTIs, while *ABCC2* polymorphisms conferred risk of intolerance to protease inhibitors containing regimens.

To date, there is no data regarding NRTIs transport through OATP transporters. In fact, OATP1B1, coded by *SLCO1B1*, plays a role in protease inhibitors uptake [28] and polymorphisms in this gene are associated to variations in plasma concentrations of these drugs [23]. Indeed, the polymorphism rs10444413 was associated with intolerance to PIs in our cohort considering a 0.05 significance level (S2 Table).

The organic anion transporter polypeptide OATP2B1, coded by *SLCO2B1*, is expressed in hepatocytes [29] and apical membrane of intestinal epithelial cells [30], among other tissues. PIs were also shown to inhibit OATP2B1 activity, suggesting that they might be a target for this transporter [31]. Several reports have shown a clear role for OATP2B1 in absorption of statins and steroid hormones, especially androgens, as well as its precursors. Accordingly, SNPs at *SLCO2B1* may predict response to these compounds [32,33]. The missense variation rs12422149 has been consistently associated to worse prognosis for prostate cancer after androgen-deprivation therapy [33]. Differential patterns of hormones absorption might indirectly influence susceptibility to lipid disorders, which are commonly observed in response to ARVs such as stavudine. In addition, since statins are commonly used in the management of lipid disorders associated to HIV infection and ARVs use [34], SNPs at *SLCO2B1* may also limit the effectiveness of this treatment in individuals undergoing antiretroviral therapy. Further analyses are required to test these hypotheses and define the role of OATP2B1 in response to antiretroviral therapy.

Beyond the classical candidate *ABCB1* (P-glycoprotein), the ABCC transporters, or multidrug resistance-associated proteins (MRP), also promote the efflux of many drugs from cells. The MRP2 transporter, encoded by *ABCC2*, has been consistently associated to the efflux of protease inhibitors from hepatocytes and peripheral blood mononuclear cells [35–38]. In the present study, the most prominent effect was observed for the SNP rs4148396, which was associated with intolerance to PIs even after Bonferroni adjustment (OR_CT/TT_ = 2.64; p = 0.00009; Table 3). This SNP, as well as rs3740066 were previously associated to neurotoxicity after treatment with the adjuvant FOLFOX4 in patients with colorectal cancer [39]. Besides *ABCC2*, SNPs in other two ABC transporters were also associated to intolerance to PIs (*ABCC4* and *ABCG2*, S2 Table), reinforcing the role of efflux transporters in adverse reactions to this class of drugs [12].

SNPs at *ABCC2* have been consistently associated to tenofovir-induced tubulopathy [40,41]. Moreover, this transporter is also inhibited by NRTIs and NNRTIs [42], which may increase the risk of drug-drug interactions in patients undergoing cART. Notably, SNPs in this gene were also associated to intolerance in comparisons including all antiretrovirals at a 0.05 significance level (S2 Table).

We were unable to detect classical associations such as *CYP2B6* and response to efavirenz and *ABCB1* SNPs and response to protease inhibitors. A limitation of our study was the inability to perform case-control analyses specific for each drug. In this case, for example, the inclusion of individuals with intolerance to nevirapine may have diluted the effect of *CYP2B6* SNPs, which is more prominent for efavirenz response. Another possibility is the outcome under investigation, which was intolerance instead of a clearly defined adverse reaction. As a consequence, we would have limited power to detect subtle or specific effects. More specifically, a reduced number of individuals exhibiting central nervous system reactions to efavirenz among the cases of intolerance to NNRTIs might also explain the lack of association with *CYP2B6* gene. Nevertheless, we cannot rule out the possibility that these effects are not valid in our population. The effect of *CYP2B6* and *ABCB1* SNPs are dependent on genetic background [43]. In this study, the genetic background was determined using a panel of ancestry informative SNPs previously validated for the Brazilian population [27], and no associations were found even after adjustment for genetic ancestry.

In Brazil, the treatment is provided to all HIV+ individuals free of charge and a National Network for HIV Genotyping was implemented to detect HIV resistance and avoid virologic failure [26]. However, just a few studies were conducted to determine the role of host genetics in treatment response. The number of people undergoing cART is expected to increase in the next few years due to new policies that recommend starting treatment at the time of diagnosis to prevent transmission. In addition, the increase in life expectancy of HIV+ individuals will also increase the possibility of drug-drug interactions due to polypharmacy. In this scenario, it is crucial to determine the distribution of ADME markers in our population.

To our knowledge, this is the largest study conducted to describe the role of ADME genes in response to antiretroviral therapy among Brazilians until today, considering the number of candidate genes and polymorphisms. In the future, this information may help to define a panel of markers that may be used along with clinical follow up and help clinicians to manage the different profiles of response to cART using personalized regimens.

## Supporting Information

S1 Table Candidate genes and SNPs selected for case-control analysis. (XLSX)

S2 Table Results of statistically significant associations between candidate SNPs and intolerance to the different antiretroviral classes. (DOCX)
