# Supplementary material for: Single Nucleotide Polymorphisms in Cellular Drug Transporters Are Associated with Intolerance to Antiretroviral Therapy in Brazilian HIV-1 Positive Individuals
Source: PLoS One. 2016 Sep 20;11(9):e0163170. doi: 10.1371/journal.pone.0163170 (PMC5029869; doi:10.1371/journal.pone.0163170)
Supplement: S2 Table — (DOCX) [file pone.0163170.s002.docx]

**S2 Table: Results of statistically significant associations between candidate SNPs and intolerance to the different antiretroviral classes.**

| **Gene symbol** | | **SNP** | **Genotype** | **Controls ^a^** | **Cases ^a^** | **OR (_95%_CI; p) ^b^** |
| --- | --- | --- | --- | --- | --- | --- |
| **All ARVs analyses** | | |  |  |  |  |
| *ABCC2* | | rs717620 | GG | 313 (0.8) | 242 (0.71) | reference |
|  | |  | GA/AA | 80 (0.20) | 100 (0.29) | 1.51 (1.07 - 2.13; p = 0.019) |
|  | | rs2804400 | GG | 182 (0.45) | 126 (0.35) | reference |
|  | |  | GA/AA | 219 (0.55) | 232 (0.65) | 1.44 (1.07 - 1.94; p = 0.017) |
|  | | rs2804398 | TT | 202 (0.50) | 148 (0.41) | reference |
|  | |  | TA/AA | 201 (0.50) | 209 (0.59) | 1.37 (1.02 - 1.83; p = 0.03) |
|  | | rs4148396 | CC | 204 (0.50) | 146 (0.41) | reference |
|  | |  | CT/TT | 201 (0.50) | 213 (0.59) | 1.42 (1.06 - 1.90; p = 0.017) |
| *ABCC3* | | rs12451302 | GG | 110 (0.27) | 130 (0.36) | reference |
|  | |  | GT/TT | 292 (0.73) | 228 (0.64) | 0.64 (0.47 - 0.88; p = 0.005) |
| *ABCC4* | | rs16950650 | CC | 346 (0.86) | 330 (0.92) | reference |
|  | |  | CT/TT | 58 (0.14) | 28 (0.08) | 0.56 (0.35 - 0.92; p = 0.018) |
| *ABCG2* | | rs4148152 | AA | 332 (0.82) | 318 (0.89) | reference |
|  | |  | AG/GG | 73 (0.18) | 40 (0.11) | 0.56 (0.37 - 0.85; p = 0.005) |
| *CYP3A5* | | rs10249369 | AA | 366 (0.90) | 312 (0.87) | reference |
|  | |  | AG/GG | 39 (0.10) | 47 (0.13) | 1.74 (1.08 - 2.78; p = 0.021) |
| *NR1I2* | | rs6785049 | GG | 172 (0.43) | 112 (0.31) | reference |
|  | |  | GA/AA | 232 (0.57) | 245 (0.69) | 1.47 (1.07 - 2.01; p = 0.016) |
| *UGT1A3* | | rs4124874 | CC | 163 (0.40) | 104 (0.29) | reference |
|  | |  | CA/AA | 242 (0.60) | 255 (0.71) | 1.54 (1.13 - 2.09; p = 0.006) |
|  | | rs4399719 | GG | 163 (0.40) | 108 (0.30) | reference |
|  | |  | GT/TT | 241 (0.60) | 251 (0.70) | 1.46 (1.07 - 1.98; p = 0.016) |
|  | | rs3755319 | GG | 143 (0.35) | 88 (0.25) | reference |
|  | |  | GT/TT | 261 (0.65) | 270 (0.75) | 1.60 (1.16 - 2.20; p = 0.004) |
| **NRTIs analyses** | | |  |  |  |  |
| *ABCC1* | | rs212086 | GG | 306 (0.76) | 91 (0.65) | reference |
|  | |  | GA/AA | 95 (0.24) | 48 (0.35) | 1.65 (1.09 - 2.52; p = 0.021) |
|  | | rs2299670 | AA | 99 (0.25) | 55 (0.40) | reference |
|  | |  | AG/GG | 302 (0.75) | 84 (0.60) | 0.55 (0.36 - 0.84; p = 0.006) |
|  | | rs4148350 | GG | 332 (0.83) | 127(0.91) | reference |
|  | |  | GT/TT | 68 (0.17) | 13 (0.09) | 0.52 (0.27 - 0.97; p = 0.030) |
| *ABCC2* | | rs2804398 | TT | 201 (0.50) | 53 (0.38) | reference |
|  | |  | TA/AA | 199 (0.50) | 87 (0.62) | 1.60 (1.07 - 2.37; p = 0.020) |
|  | | rs2804400 | GG | 181 (0.45) | 44 (0.31) | reference |
|  | |  | GA/AA | 217 (0.55) | 97 (0.69) | 1.73 (1.14 - 2.61; p = 0.009) |
|  | | rs7080681 | GG | 364 (0.91) | 136(0.97) | reference |
|  | |  | GA/AA | 36 (0.09) | 4 (0.03) | 0.34 (0.12 - 0.97; p = 0.023) |
| *APOC3* | | rs4520 | CC | 185 (0.46) | 81 (0.58) | reference |
|  | |  | CT/TT | 216 (0.54) | 59 (0.42) | 0.61 (0.41 - 0.91; p = 0.014) |
| *CYP3A5* | | rs10249369 | AA | 364 (0.91) | 119 (0.84) | reference |
|  | |  | AG/GG | 38 (0.09) | 22 (0.16) | 2.19 (1.21 - 3.97; p = 0.011) |
| *NR1I2* | | rs6785049 | GG | 171 (0.43) | 40 (0.29) | reference |
|  | |  | GA/AA | 230 (0.57) | 100 (0.71) | 1.69 (1.10 - 2.61; p = 0.016) |
| *SLC22A2* | | rs316003 | TT | 150 (0.37) | 72 (0.51) | reference |
|  | |  | TC/CC | 252 (0.63) | 69 (0.49) | 0.61 (0.41 - 0.90; p = 0.014) |
| *SLCO1B3* | | rs7962265 | AA | 169 (0.42) | 41 (0.29) | reference |
|  | |  | AG/GG | 232 (0.58) | 99 (0.71) | 1.61 (1.05 - 2.47; p = 0.026) |
| *SLCO2B1* | | rs12422149 | GG | 296 (0.74) | 119 (0.84) | reference |
|  | |  | GA/AA | 106 (0.26) | 22 (0.16) | 0.50 (0.30 - 0.84; p = 0.006) |
|  | | rs1676885 | AA | 298 (0.74) | 88 (0.63) | reference |
|  | |  | AG/GG | 104 (0.26) | 52 (0.37) | 1.55 (1.01 - 2.36; p = 0.044) |
|  | | **rs2712816** | **GG** | **171 (0.43)** | **32 (0.23)** | **reference** |
|  | |  | **GA/AA** | **230 (0.57)** | **109 (0.77)** | **2.37 (1.51 - 3.72; p = 0.0001)** |
|  | | rs949069 | GG | 146 (0.36) | 73 (0.52) | reference |
|  | |  | GA/AA | 255 (0.64) | 68 (0.48) | 0.57 (0.38 - 0.84; p = 0.005) |
| **NNRTIs analyses** | | |  |  |  |  |
| *ABCC3* | | rs739923 | GG | 167 (0.56) | 42 (0.42) | reference |
|  | |  | GA/AA | 129 (0.44) | 57 (0.58) | 1.77 (1.11 - 2.81; p = 0.015) |
|  | | rs733392 | GG | 170 (0.57) | 43 (0.43) | reference |
|  | |  | GA/AA | 126 (0.43) | 56 (0.57) | 1.72 (1.08 - 2.72; p = 0.021) |
| *ABCC4* | | rs899494 | GG | 212 (0.72) | 58 (0.59) | reference |
|  | |  | GA/AA | 84 (0.28) | 40 (0.41) | 1.79 (1.11 - 2.88; p = 0.018) |
| *ABCG2* | | rs17731538 | GG | 236 (0.80) | 64 (0.65) | reference |
|  | |  | GA/AA | 61 (0.20) | 35 (0.35) | 2.07 (1.25 - 3.41; p = 0.005) |
| *CYP3A4* | | rs12333983 | TT | 116 (0.39) | 55 (0.56) | reference |
|  | |  | TA/AA | 180 (0.61) | 44 (0.44) | 0.53 (0.32 - 0.88; p 0.013) |
| *NR1I2* | | rs6785049 | GG | 132 (0.44) | 30 (0.31) | reference |
|  | |  | GA/AA | 165 (0.56) | 68 (0.69) | 1.73 (1.04 - 2.87; p = 0.030) |
| *SLC22A2* | | rs17588242 | TT | 209 (0.70) | 55 (0.56) | reference |
|  | |  | TC/CC | 88 (0.30) | 44 (0.44) | 1.83 (1.13 - 2.95; p = 0.014) |
|  | | rs316019 | GG | 220 (0.74) | 84 (0.86) | reference |
|  | |  | GT/TT | 77 (0.26) | 14 (0.14) | 0.48 (0.26 - 0.90; p = 0.015) |
| *UGT1A3* | | rs4124874 | CC | 129 (0.43) | 28 (0.28) | reference |
|  | |  | CA/AA | 168 (0.57) | 71 (0.72) | 1.88 (1.14 - 3.10; p = 0.011) |
|  | | rs4399719 | GG | 129 (0.43) | 28 (0.28) | reference |
|  | |  | GT/TT | 168 (0.57) | 71 (0.72) | 1.88 (1.14 - 3.10; p = 0.011) |
|  | | rs3755319 | GG | 117 (0.40) | 24 (0.24) | 0.00893 |
|  | |  | GT/TT | 179 (0.60) | 74 (0.76) | 1.96 (1.17 - 3.29; p = 0.009) |
| **PIs analyses** | | |  |  |  |  |
| *ABCC2* | rs2073337 | | AA | 88 (0.43) | 30 (0.26) | 0.00149 |
|  |  | | AG/GG | 115 (0.57) | 85 (0.74) | 2.24 (1.34 - 3.73; p = 0.001) |
|  | rs2804398 | | TT | 108 (0.54) | 39 (0.34) | reference |
|  |  | | TA/AA | 93 (0.46) | 75 (0.66) | 2.25 (1.38 - 3.66; p = 0.001) |
|  | rs2804400 | | GG | 95 (0.47) | 34 (0.30) | reference |
|  |  | | GA/AA | 106 (0.53) | 81 (0.70) | 2.07 (1.25 - 3.43; p = 0.004) |
|  | rs3740066 | | GG | 115 (0.57) | 48 (0.42) | reference |
|  |  | | GA/AA | 88 (0.43) | 67 (0.58) | 1.82 (1.13 - 2.94; p = 0.014) |
|  | **rs4148396** | | **CC** | **110 (0.54)** | **36 (0.31)** | **reference** |
|  |  | | **CT/TT** | **93 (0.46)** | **79 (0.69)** | **2.64 (1.61 - 4.33; p = 0.00009)** |
| *ABCC4* | rs16950650 | | CC | 171 (0.84) | 109 (0.95) | reference |
|  |  | | CT/TT | 32 (0.16) | 6 (0.05) | 0.34 (0.13 - 0.85; p = 0.012) |
| *ABCG2* | rs13120400 | | TT | 147 (0.72) | 64 (0.56) | reference |
|  |  | | TC/CC | 56 (0.28) | 51 (0.44) | 1.99 (1.23 - 3.24; p = 0.005) |
| *SLCO1B1* | rs10444413 | | TT | 139 (0.72) | 92 (0.81) | reference |
|  |  | | TC/CC | 54 (0.28) | 21 (0.19) | 0.52 (0.29 - 0.93; p = 0.025) |

All SNPs represented were associated to intolerance under dominant and codominant models (p < 0.05) before adjustment for covariates. Associations that remained statistically significant after Bonferroni adjustment (alpha=0.0002) are represented in bold. OR = odds ratio; CI = confidence interval; ARVs=antiretrovirals; NRTIs=nucleoside reverse transcriptase inhibitors; NNRTIs=non-nucleoside reverse transcriptase inhibitors; PIs=protease inhibitors.

^a^ results are shown as N (frequency).

^b^ results are adjusted for gender and genetic ancestry.
